# Beyond the hockey stick: Climate lessons from the Common Era

**DOI:** 10.1073/pnas.2112797118

**Published:** 2021-09-24

**Authors:** Michael E. Mann

**Affiliations:** ^a^Department of Meteorology and Atmospheric Science, The Pennsylvania State University, University Park, PA 16802

**Keywords:** Common Era, hockey stick, climate change, paleoclimatology

## Abstract

More than two decades ago, my coauthors, Raymond Bradley and Malcolm Hughes, and I published the now iconic “hockey stick” curve. It was a simple graph, derived from large-scale networks of diverse climate proxy (“multiproxy”) data such as tree rings, ice cores, corals, and lake sediments, that captured the unprecedented nature of the warming taking place today. It became a focal point in the debate over human-caused climate change and what to do about it. Yet, the apparent simplicity of the hockey stick curve betrays the dynamicism and complexity of the climate history of past centuries and how it can inform our understanding of human-caused climate change and its impacts. In this article, I discuss the lessons we can learn from studying paleoclimate records and climate model simulations of the “Common Era,” the period of the past two millennia during which the “signal” of human-caused warming has risen dramatically from the background of natural variability.

## Significance

I review the significant developments, current challenges, and prospective future directions in the subfield of paleoclimatology of the Common Era since the publication of the now iconic “hockey stick” curve by the author and collaborators more than two decades ago, with a focus on how paleoclimate information can inform our understanding of the impact of human-caused climate change.

Clearly, there is a cautionary tale told by the hockey stick curve in the unprecedented warming that we are causing, but the lessons from the paleoclimate record of the Common Era (CE) go far beyond that. What might we infer, for example, about the role of dynamical mechanisms relevant to climate change impacts today from their past responses to natural drivers? Examples are the El Niño phenomenon, the Asian summer monsoon, and the North Atlantic Ocean “conveyor belt” circulation. Are there potential “tipping point” elements within these climate subsystems? How has sea level changed in past centuries, and what does it tell us about future coastal risk? Are there natural long-term oscillations, evident in the paleoclimate record, that might compete with human-caused climate change today? Can we assess the “sensitivity” of the climate to ongoing human-caused increases in greenhouse gas concentrations from examining how climate has responded to natural factors in the past? Also, can better estimates of past trends inform assessments of how close we are to critical “dangerous” warming thresholds? In this article, I seek to address such questions and offer thoughts about ways forward to more confident answers.

## A Longer, Sturdier Hockey Stick

**Fig. 1.**
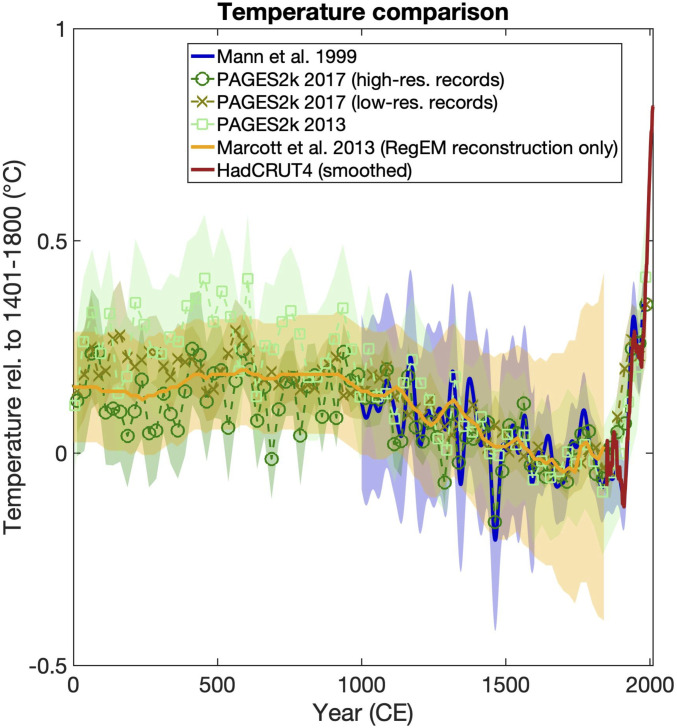
Comparison of temperature reconstructions spanning the CE including the original Mann et al. (2) hockey stick reconstruction (1, 2) and its 95% uncertainty range and several different versions of the PAGES2k (Past Global Changes Last Two Millennium initiative) reconstruction (4) and uncertainty range, as well as the lower-resolution reconstruction of Marcott et al. (5) and its uncertainties. The smoothed Hadley Center and Climatic Research Unit Surface Temperature Product version 4 (HadCRUT4) instrumental global temperature series is shown for comparison. RegEM refers to a statistical reconstruction based on the Regularized Expectation-Maximization method.

In the two decades since the original “hockey stick” works of Mann et al. [1998 (1) and 1999 (2), often referred to as “MBH98” and “MBH99,” corresponding to authors Mann, Bradley, and Hughes, respectively], which extended back to 1000 CE, far more paleoclimate data have become available, more sophisticated methods have been developed and applied to these data, and longer reconstructions have been achieved using lower-resolution but considerably longer paleoclimate proxy records. The net result is a veritable “hockey league” (3, 4)—dozens of independent studies that come to similar conclusions and a longer, sturdier hockey stick (Fig. 1). Updated reconstructions show the recent warming to be anomalous in an even longer-term context, at least the past two millennia and more tentatively, the past 20,000 y (5, 6), than originally concluded two decades ago by Mann et al. (2). Studies using climate models driven by estimated natural (volcanic and solar) and anthropogenic forcing demonstrate that only the latter can explain this unprecedented warming trend (3).

From the standpoint of the public climate change discourse, these conclusions are significant. They underscore the profound, unprecedented impact human activity—fossil fuel burning in particular—is having on our planet. From a scientific standpoint, however, some of the more important insights—insights that indeed motivated the original work of MBH98—involve past patterns of variability and what they can tell us about the dynamics of the climate system and the response to external drivers or “forcings.” We explore these insights in the following section.

## Dynamical Mechanisms and Responses

Many of the key impacts of climate change involve dynamical components of the climate system and their response to anthropogenic climate forcing. These include the El Niño/Southern Oscillation (ENSO), which influences weather patterns around the world, impacting western US drought and Atlantic hurricane activity among other phenomena. These include the Arctic Oscillation (AO) or closely related North Atlantic Oscillation (NAO), which impact weather patterns in North America and Eurasia. They also include the Asian summer monsoon upon which more than a billion people depend for their freshwater supply. Finally, there is the Atlantic Meridional Overturning Circulation (AMOC), often termed the “ocean conveyor belt” circulation. The AMOC delivers warm water to the high latitudes of the North Atlantic, warming neighboring regions and circulating nutrients to North Atlantic surface waters while suppressing sea level along parts of the US East Coast.

We can better understand these key dynamical components of the climate system and their potential role in climate change by studying how they have responded to past natural drivers, such as volcanic and solar radiative forcing. Moreover, we can assess whether dramatic changes in these systems are underway today by comparing recent trends against the record of the past one to two millennia.

### ENSO.

Drought in the desert Southwest, which impacts large population centers in California, Nevada, and Arizona, is modulated by ENSO. While the influence varies from one event to the next, El Niño years tend to be wetter than normal, and La Nina years tend to be drier than normal. ENSO also impacts Atlantic hurricane activity, with El Niño years less active and La Nina years more active than normal. Any changes in ENSO mean state and variability could have profound impacts on these and other phenomena impacting North and South America, Africa, Australia, Indonesia, and other regions influenced by ENSO.

State-of-the-art climate models such as those used in the most recent Intergovernmental Panel on Climate Change (IPCC) Fifth Assessment Report (AR5) assessment, however, provide limited guidance. They display a large range in the response of the tropical Pacific mean state to anthropogenic greenhouse forcing. Moreover, the models, on the whole, are inconsistent with the observations; the models exhibit a trend toward an El Niño–like mean state, with a decreased contrast between the western Pacific warm pool and eastern Pacific “cold tongue,” while the observations show a neutral or even opposite, La Nina–like trend over the past half-century. Seager et al. (7) argue that this failure for models to produce the observed response to heating is a consequence of a biased mean state in the models, associated with too strong upwelling in the cold tongue in the eastern equatorial Pacific. They warn that this may bias climate model projections in the many regions sensitive to tropical Pacific sea surface temperature (SST) forcing.

The dynamical mechanisms in question relate to earlier work by Clement and colleagues (8, 9), who argued that the upwelling of cold waters and shallow thermocline in the eastern equatorial Atlantic oppose radiatively forced warming in the eastern equatorial Pacific as the western equatorial Pacific continues to warm. The Bjerknes feedbacks amplify this forced response, creating stronger trade winds and a stronger zonal SST gradient (i.e., an overall “La Nina”–like relative pattern of SST change). Recent work yields a more nuanced picture, suggesting a transient tug-of-war between these dynamical responses and the thermodynamic response of a warming thermocline, with a net response that may be timescale dependent (10–14). The importance of the dynamical response to forcing nonetheless appears to remain relevant on the multidecadal to centennial timescales of interest.

Multiproxy reconstructions of surface temperature patterns spanning the past millennium based on tree rings, corals, lake sediments, ice cores, and other proxy sources seem consistent with such a dynamical response, displaying a La Nina–like cooling in the eastern equatorial Pacific (15) and a pattern of a dry desert Southwest United States (16) and wet Pacific Northwest United States (17) that is consistent with a La Nina–like state during the early part (AD 1000 to 1400) of the past millennium. Consistent with the hypothesized dynamical response, that state coincides with a period of anomalous positive (high-solar, low-volcanic) natural radiative forcing. The La Nina–like pattern is not reproduced in global coupled climate model simulations of the past millennium (8), which could be due to uncertainties and biases in the proxy records available that far back in time and/or biases in the models. As noted earlier, current generation climate models do not reproduce the observed historical trend of little or no warming in the eastern equatorial Pacific. The paleoclimate record of the past millennium, in that sense, seems to reinforce the notion that models are not getting certain important dynamical response to forcing right and may be underestimating key climate change impacts such as aridification in the western United States and heightened hurricane activity in the tropical Atlantic.

One important contributor to the medieval La Nina pattern is the relative absence of volcanic eruptions during the earlier centuries of the past millennium. A number of observational (18) and modeling (19, 20) studies indicate a tendency for an El Niño–like response to volcanic radiative forcing, consistent with the hypothesized role of the Bjerkness feedbacks. One recent study based on a long coral record from the central equatorial Pacific (21) argues against such a response. However, others (20) have noted that a coral proxy responding to local SST changes in the central equatorial Pacific might not detect a response at all, while remotely located coral or tree-ring proxies, such as those used in large-scale climate reconstructions (15), might better detect an El Niño–like response. Additional high-resolution proxy records from key ENSO-ensitive regions spanning the past millennium might shed further light on this puzzle. At a time when one impacted region—the desert Southwest United States—is experiencing droughts that are unprecedented in at least 1,200 y (22), this is an important puzzle to solve.

### AO/NAO.

Another intriguing dynamical response to forcing involves the AO/NAO, a pattern of variation in the winter storm track from year to year that is especially prominent over the North Atlantic sector and impacts winter temperatures and precipitation over a large part of North America and Eurasia. Multiproxy reconstructions and model simulations suggest that the relative cold of certain regions like Europe during the so-called “Little Ice Age” (e.g., fifteenth to nineteenth centuries) and conversely, the relative warmth of those regions during the Medieval era (eleventh to fourteenth centuries) are consistent with negative and positive AO/NAO-like patterns, respectively, during those time intervals (15).

This response appears to be driven by the interaction between solar ultraviolet radiation and lower stratospheric/upper tropospheric atmospheric dynamics that leads to a negative AO/NAO pattern during periods of low solar irradiance (e.g., the Little Ice Age) and conversely, a positive AO/NAO pattern during Medieval times. Mann et al. (15) show that a simulation of the past millennium with a model that includes interactive ozone photochemistry reproduces the pattern in the multiproxy reconstructions, while a simulation with a model that lacks these processes does not. The absence of interactive ozone photochemistry in the vast majority of last millennium intercomparisons, e.g., the Coupled Model Intercomparison Project Phase 5 (CMIP5), is a severe limitation of the ability of those models to capture key regional climate responses (23), one that should be addressed in future such intercomparisons.

### South Asian Summer Monsoon.

Precipitation tied to the South Asian summer monsoon (SASM) provides fresh water for a large population in South Asia, and its potential future behavior under climate change is a matter of considerable attention. While we can learn something about this important component of the climate system from studying its response to past natural radiative forcing, we must also be aware of the limitations and caveats involved.

The SASM is characterized by a largely meridional–vertical circulation with rising motion over the Indian subcontinent driven by differential solar heating and orographic lifting and descending motion over the Indian Ocean. It is sometimes equated with the precipitation that occurs over land as a consequence of this circulation. However, the monsoonal circulation and rainfall need not covary. In what has sometimes been referred to as the “wind–precipitation paradox” (24, 25), moistening of the midtroposphere under, for example, greenhouse warming leads to increased rainfall but stabilization of the midtroposphere in the sinking region of the SASM, inhibiting the monsoonal overturning circulation. A divergence is thus seen between SASM circulation and SASM-produced precipitation during the twentieth century and in future projections (24, 25). Recent, high-resolution model simulations demonstrate that the two quantities are in fact essentially decoupled; it is possible to have strong monsoon winds with no rain or monsoon rain with weak meridional winds (26).

In an analysis of a simulation of the past millennium, Fan et al. (27) found only moderate covariation between the SASM circulation and SASM rainfall during the preindustrial period and a marked decoupling during the modern era, where the SASM circulation weakens but SASM precipitation remains roughly constant. Such findings present a challenge for the interpretation of proxy reconstructions of the SASM over the past millennium and potentially explain the divergence seen among various purported SASM proxies, some (e.g., tree rings) being proxies for rainfall while others are proxies for wind speed and direction (3, 19, 28).

There are additional challenges in drawing conclusions about the SASM from both simulations and proxy data of the past millennium. There is an important influence of ENSO on the SASM, with El Niño (La Nina) leading to a tendency for a weakened (strengthened) monsoonal circulation. Fan et al. (27) observe a significant weakening of the SASM in response to past volcanic forcing, consistent with reduced surface solar heating that induces a reduction in overturning circulation. However, the dynamical response of ENSO to radiative forcing discussed earlier is not evident in most coupled model simulations, including the one they analyzed. This response should lead to further weakening of the SASM owing to an El Niño–like response to volcanic forcing. A further complication is the potential role of Indo-Pacific coupling that is known to impact the SASM but is not well captured in models during the modern era (29), and appears to have exhibited substantial variability over the past millennium (30).

Future proxy-based work should adopt a more systematic approach to defining past changes in the SASM, distinguishing in particular between proxies of circulation and proxies of precipitation. Future modeling work should employ higher-resolution, next generation climate model simulations where SASM dynamics are well resolved and where key dynamical mechanisms, such as the response of ENSO to radiative forcing, are more faithfully represented.

### The AMOC.

One of the most significant potential dynamical responses to anthropogenic climate forcing involves the behavior of the AMOC/ocean conveyor belt, a thermohaline overturning circulation that is driven by the sinking of cold, fresh water in high-latitude regions of the North Atlantic. While there has been much focus on the role of freshwater input and AMOC weakening during the last glacial termination and the so-called 8.2-ka event during the Early Holocene, there has been less focus on the behavior of the AMOC during the CE. Rahmstorf et al. (31), however, estimated changes in AMOC over the past millennium using both an AMOC index derived from multiproxy-based North Atlantic SST reconstructions (15) and coral proxy δ^15^N data that serve as tracers of Labrador slope water. They found an anomalous decrease in the strength of the AMOC over the past century in the context of the past millennium. More recent work by Caesar et al. (32) using marine sediment silt data and foraminifera proxy data provide longer-term, additional evidence, suggesting that the AMOC slowdown over the past century is unprecedented since at least AD 400.

This finding is significant because AMOC collapse constitutes one of the potential tipping point responses to anthropogenic climate forcing. As climate models do not predict a substantial weakening until the late twenty-first century, the fact that weakening may have already occurred during the twentieth century suggests AMOC collapse could be proceeding faster than scheduled, perhaps due to earlier than expected freshwater input from Greenland melt (31). While direct AMOC observations show conflicting trends (33), one recent study (34), based on a variety of complementary metrics, argues that AMOC collapse does appear underway.

Potential impacts of AMOC collapse include decreased marine productivity in the North Atlantic, accelerated sea-level rise along parts of the US East Coast (arising from the geostrophic balance maintaining the northward current system), and the potential for greater tropical North Atlantic warming and increased Atlantic hurricane activity (both of which are discussed in the subsequent section). It is thus of vital importance to address the current discrepancies among models and observations. One current limitation is that interactive ice sheet meltwater coupling is not incorporated into multimodel climate change experiments (e.g., CMIP5 and CMIP6). Including such processes in future modeling experiments should allow for more confident conclusions. Meanwhile, a “future-proof” network based on existing and new AMOC observational approaches could better constrain historical trends (33).

## Sea-Level Rise and Tropical Cyclones

Climate change poses twin threats to coastal settlement in the form of sea-level rise and more intense tropical cyclones (TCs). Paleoclimate data and model simulations of the CE can inform our understanding of these threats and place them in a longer-term context.

Reconstructions of landfalling Atlantic TCs from coastal overwash deposits spanning the past two millennia suggest that the increase in basin-wide Atlantic TC activity over the past several decades is unusual, but not necessarily unprecedented, over that time frame (35). Comparison with a statistical model of Atlantic TC activity driven by proxy-reconstructed (15) indices of ENSO, tropical Atlantic SST, and the NAO predicts a period of high activity during the Medieval (associated with a warm tropical Atlantic and a prevalent La Nina–like state) that is matched by the overwash deposit evidence, but there is a discrepancy during the fifteenth century where the overwash deposits indicate a period of high activity that is not matched by the proxy climate index–driven statistical model.

Synthetic long-term hurricane datasets derived using downscaling approaches applied to coupled model millennium simulations of the past millennium have been used both to address statistical sampling issues (36) and examine long-term climate/TC relationships (36). Kozar et al. (36) analyzed downscaled synthetic TCs from an early forced millennial simulation of the National Center for Atmospheric Resesarch (NCAR) Climate System Model (CSM) 1.4 coupled model, concluding that proxy composites of overwash deposits from a modest set of locations along the US East Coast, Gulf Coast, and Caribbean are reasonably representative of basin-wide TC activity, although multidecadal periods of divergence, such as observed during the fifteenth century, are consistent with expected sampling fluctuations. These conclusions are supported by additional work by Reed et al. (37) examining downscaling results from the CMIP5 “Last Millennium” simulations. These simulations show only a weak long-term relationship between tropical Atlantic SST and measures of integrated TC intensity (e.g., the power dissipation index), suggesting that the strong relationship between the two quantities during the modern era (38) might be specific to modern anthropogenic warming in recent decades and not generalizable. More confident assessments of long-term basin-wide trends should be possible as additional long-term paleohurricane records are recovered (39, 40).

Sea-level reconstructions based on coastal deposits indicate that the current rate of sea-level rise is unprecedented over the past two millennia (41, 42). The combination of rising sea level and more intense hurricanes that arise in downscaled historical simulations (43) has led to vastly reduced return periods for Superstorm Sandy–like storm surges for New York City. Garner et al. (44) find that the 500-y return period for a 2.25-m flood height during the preanthropogenic CE has decreased to roughly 24 y in the anthropogenic era. They find that a combination of projected future sea-level rise and further intensification of hurricanes would likely yield permanent inundation for New York City under business as usual emissions, although a tendency for continued poleward shift in hurricane paths with climate change that has been noted in historical and paleoclimate observations (45, 46) tends to mitigate risk for New York City at the expense of increased risk farther north (e.g., Boston).

## Internal Climate Oscillations

The analysis of paleoclimate data by Mann et al. (1, 2) that led to the hockey stick reconstruction of Northern Hemisphere average temperature was an outgrowth of earlier work by Mann et al. (47) analyzing networks of multiproxy data to assess evidence for natural long-term climate oscillations. Evidence of an apparent spatiotemporal mode of large-scale surface temperature variability emphasizing the North Atlantic basin combined with evidence of a similar mode of climate variability in control simulations of early generation–coupled ocean–atmosphere models (48) led to the notion of the “Atlantic Multidecadal Oscillation” (AMO)—a term originally coined by Mann in an interview with Richard Kerr of *Science* magazine (49).

The notion that a natural, internal oscillation with a multidecadal (50- to 70-y period) timescale might be responsible for an array of climate trends, including tropical Atlantic warming and increases in Atlantic TC activity, has since become widespread. A body of work over the past decade analyzing observations and climate model simulations, however, strongly calls into question whether such a mode of internal climate variability even exists (ref. 49 has a review).

Mann et al. (49) showed that control simulations of state-of-the-art (CMIP5) climate models do not produce any consistent evidence for an internal AMO-like oscillation (or an interdecadal “Pacific Decadal Oscillation”). Indeed, they find they show no evidence of oscillatory variability other than the interannual ENSO phenomenon. An apparent ∼50-y oscillatory signal in the instrumental surface temperature record is reproduced from historical climate model simulations and seen to be an artifact of the competition between long-term greenhouse warming and the more recent decrease in sulfate aerosol cooling in the late twentieth century, rather than a natural long-term climate oscillation.

**Fig. 2.**
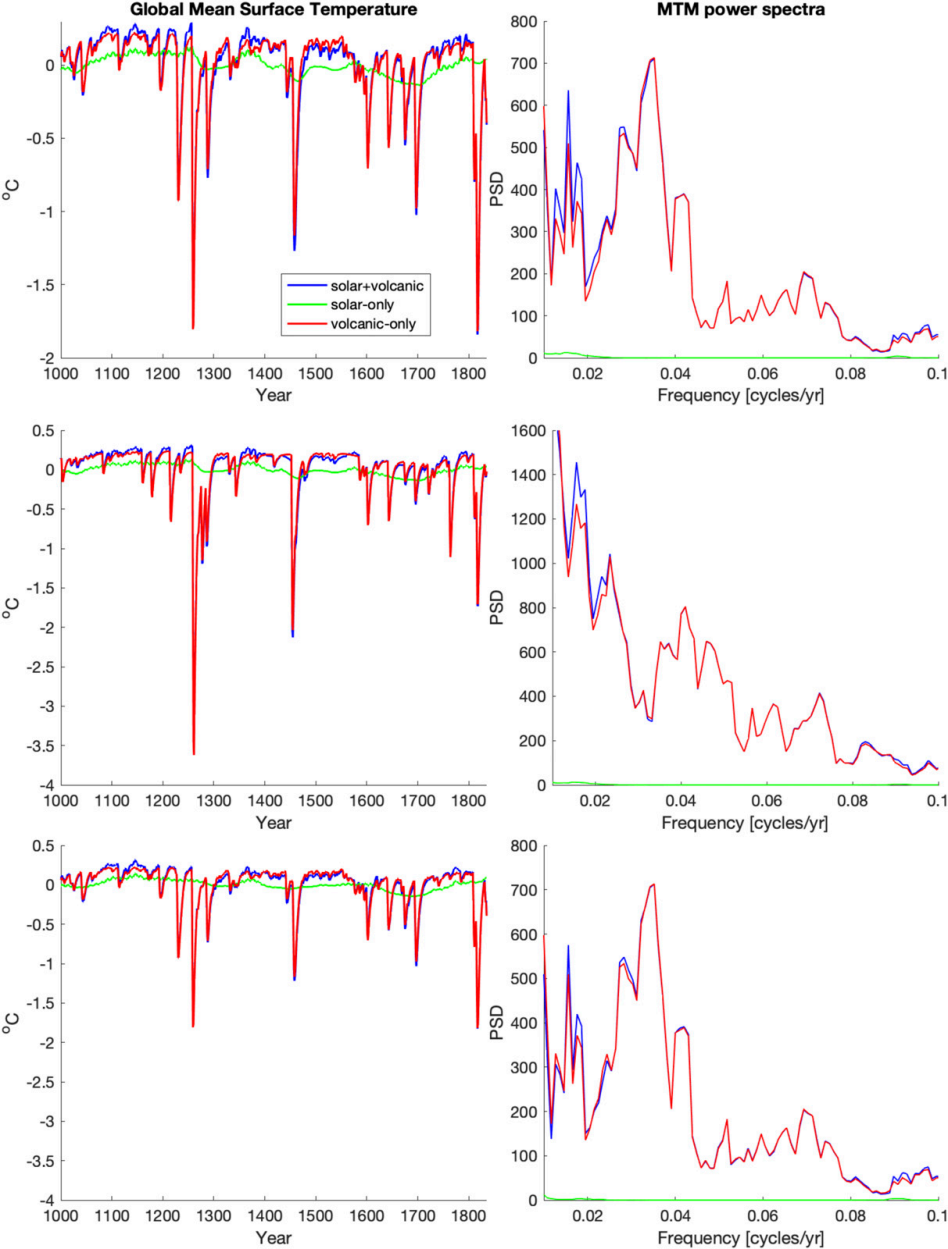
Energy Balance Model (EBM) simulations and associated power spectral density (PSD) estimates (from ref. 51). Shown are (*Left*) global mean surface temperature anomaly series from 1000 to 1835 CE and (*Right*) corresponding MTM (Multitaper Method) power spectra using both solar and volcanic forcing (blue), solar only (green), and volcanic only (red). Forcings used correspond to CEA volcanic series and SBF solar series (*Top*), GRA (Gao et al.) volcanic series and SBF (Steinhilber et al.) solar series (*Middle*), and CEA (Crowley and Untermann) volcanic series and VSK (Vieira et al.) solar series (*Bottom*). [See ref. 51 for full details.]

Increasingly, the prevailing view is that decadal and longer timescale internal variability is indistinguishable from colored noise (50). One seeming contradiction with that interpretation, however, is the aforementioned evidence of interdecadal and multidecadal spectral peaks in the analysis of paleoclimate proxy data from past centuries (47). Focusing on the putative ∼40- to 60-y AMO signal, Mann et al. (51) recently showed that multidecadal spectral peaks evident in analyses of climate model simulations of the past millennium (CMIP5 Last Millennium experiments) are a consequence of the coincidental multidecadal pacing by explosive volcanism in past centuries, explaining why these peaks are seen in the (forced) CMIP5 Last Millennium simulations but not control simulations of the very same models. Indeed, they show that these spectral peaks are evident in simple zero-dimensional energy balance models forced by estimated past volcanic radiative forcing (Fig. 2). Recent work by Waite et al. (52) comes to similar conclusions based on a comparison of sclerosponge proxy data and CMIP5 Last Millennium simulations.

The best available evidence today thus argues against the existence of internal interdecadal and multidecadal climate oscillations, casting doubt on claims that an AMO oscillation is responsible for increases in tropical Atlantic SST or Atlantic hurricane activity and casting doubt on prospects for long-term predictability of internal interdecadal and multidecadal climate variability. Further analysis of paleoclimatic data and direct comparisons with forced model simulations should allow for further validation of the recent conclusions by Mann et al. (51) regarding the apparent volcanic origin of AMO-like variability in past centuries.

## Climate Sensitivity

Paleoclimate plays an important role in informing assessments of the warming effect of an increase in greenhouse gas concentrations. That effect can be measured in terms of the transient climate response (TCR), defined as the warming at a given point in time when a doubling of the concentration of carbon dioxide in the atmosphere (equivalent to a roughly 3.7-W/m^2^ radiative forcing) has been achieved. Most paleoclimate studies, however, focus on the so-called equilibrium climate sensitivity (ECS), the estimated warming that results, in equilibrium, from that same doubling. This is the “Charney” definition of climate sensitivity and accounts for “fast” feedbacks related to clouds, water vapor, ice, etc. (an alternative related quantity, known as earth system sensitivity, takes into account slow feedbacks related to ice sheet dynamics, long-term shifts in vegetation zones, etc.).

**Fig. 3.**
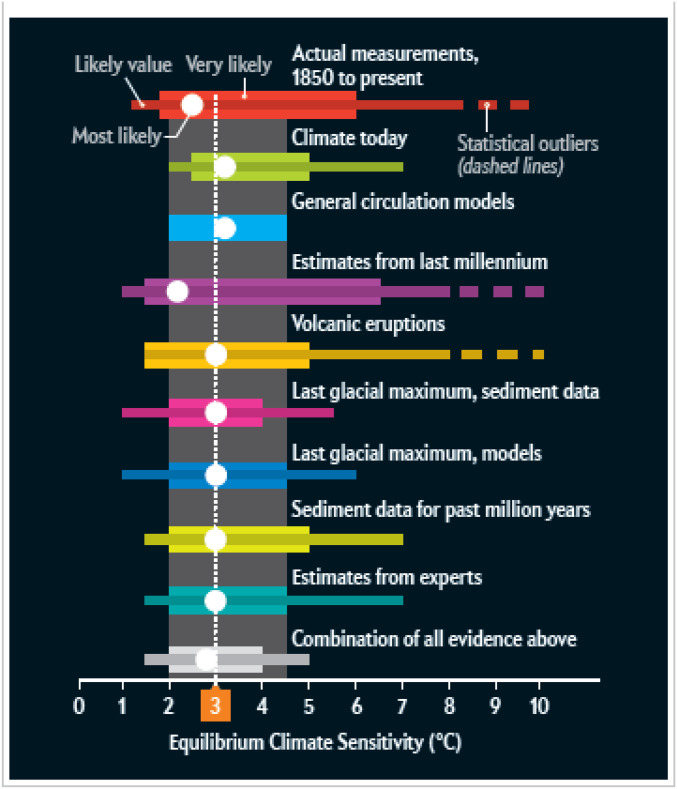
Estimates of ECS from various lines of evidence. Adapted with permission from ref. 53.

Although defined in the context of greenhouse warming, ECS is often regarded as universal enough of a quantity that it can be measured from the response of the climate to other radiative forcings, including natural solar and volcanic radiative forcing (although caveats herein are noted below). The historical record provides a relatively poor constraint on estimates of ECS owing to the shortness of the record and the fact that there are multiple competing and uncertain (particularly anthropogenic aerosol) radiative forcings over the duration of the record. ECS values lower than 1.5 °C or higher than 8.5 °C cannot be ruled out with a reasonable (68%) degree of confidence from this line of evidence alone (Fig. 3).

The “likely” range of ECS, based on a variety of lines of independent (or largely independent) evidence, is generally considered to be ∼1.5 to 5 °C (Fig. 3), with a central estimate of 3 °C. Such evidence (53, 54) includes the match of model simulations to current climatological averages, the average value of ECS as diagnosed directly from climate models, the response of the climate to volcanic eruptions, observations from the Last Glacial Maximum (LGM), geological evidence over millions of years, expert judgment, and last but not least, comparisons of paleoclimate observations and model simulations over the past millennium.

It is notable that this last line of evidence leads to the lowest estimate of ECS of all, just above 2 °C (Fig. 3), whereas the average among all lines of evidence is closer to 3 °C. In most last millennium studies, ECS estimates are typically obtained by varying the ECS value in an energy balance model simulation (where it is a simple adjustable parameter) and determining the value at which the best fit is achieved between simulated and proxy-reconstructed global or hemispheric mean temperatures. The dominant source of radiative forcing during the preindustrial CE is the cooling response to volcanic aerosol loading of the stratosphere during the years following an explosive (typically tropical) volcanic eruption. In fact, one prominent study (55) only used data back to 1270 CE to avoid the largest estimated volcanic forcing event of the past millennium (the 1258 CE eruption) where a dramatic data/model mismatch is observed. While the eruption is estimated to have given rise to a radiative forcing of about −12 W/m^2^ (roughly four times larger than the 1991 Pinatubo eruption), little or no response is seen in tree-ring temperature proxies, which make up a significant component of most proxy reconstructions of hemispheric and global mean temperature. Although this problem is highlighted by the 1258 CE discrepancy, it is hardly likely to be limited to that year.

Mann et al. (56) use a combination of model-simulated temperature over the past millennium and a simple simulation of tree-ring responses to argue that the reliance on tree line–proximal tree-ring sites in temperature reconstructions leads to both an underestimation bias in recording very large eruptions (which dominate the forced climate response prior to the industrial era) and chronological errors that accumulate back in time. This arises from the fact that very cold summers may lie below the minimal temperature threshold for growth—a problem that leads to a loss of sensitivity to cooling (correlated over large spatial regions) and the potential accumulation of chronological errors back in time (if there is no growth during a given summer season, then there is no ring recorded). This effect leads to an attenuation and smearing of the apparent response to very large eruptions that increases back in time. Those features are reproduced by the simulated tree growth response (56) and they lead to a substantial bias in estimating ECS values, yielding an estimated ECS of ∼2.0 °C when the true value is 3.0 °C (57).

While tree-ring researchers have strongly objected to these conclusions (58), there are additional lines of evidence that support them, including 1) resampling experiments that show that shifts consistent with the estimated chronological errors in age models yield surrogates with large simultaneous responses to the 1258 CE and 1815 CE eruptions consistent with the model-simulated response (56) and 2) that a realignment of specific tree-ring series consistent with the estimated chronological error range yields a much larger and sharper hemispheric mean cooling response to the 1258 CE eruptions (59).

Regardless of the source of the discrepancy, it is clear that the mismatch between the reconstructed and modeled volcanic cooling leads to an ECS underestimation bias. Analyzing the CMIP5 Last Millennium forced simulations, Schurer et al. (60) show that simply removing the few largest volcanic forcing events from model/data comparisons results in a substantially larger inferred forced response from proxy temperature reconstructions that is consistent with model simulations and the average CMIP5 model ECS value of roughly 3.2 °C. A further complication is the fact that volcanic events measure a short-term transient response to forcing that is arguably more of a measure of TCR than ECS, and there are substantial uncertainties involved in translating TCR to an equivalent ECS estimate (61).

There is a larger potential problem here, however, that goes beyond the issue of how well proxy data record past climate change. Forced climate responses during the CE are dominated by forcings (i.e., explosive volcanic eruptions) that lead to substantial cooling relative to current temperatures. This is relevant because ECS is not a universal quantity. It involves feedback processes that are in general not the same for cold and warm global climates. Cold global climates, for example, are more likely to be impacted by cryosphere responses such as ice cover–related albedo changes, while warm global climates are more likely to be impacted by carbon cycle feedbacks related to permafrost melt and methane release or warm climate threshold cloud responses (62). We are still in the process of better understanding the potential feedback processes that may arise in hothouse climates (63, 64).

This asymmetry could mean that ECS values obtained from past “cold climate” responses are not especially instructive when it comes future potential greenhouse warming. A worst case scenario could take us to CO_2_ levels not seen in tens of millions of years. Herein, we face a “catch 22.” Our most reliable paleoclimate constraints include the more recent past (e.g., the CE and the LGM), where both paleodata and relevant forcings are best known. Yet, both yield cold climate estimates of ECS. To find analogs for present greenhouse gas levels, we must go back to at least the Early Pliocene 5 Mya. To find “warm climate” analogs for greenhouse gas levels of ∼1,200 ppm CO_2_ equivalent, which we could reach by the end of the century under a worst case scenario (i.e., no substantial reductions in carbon emission), we must go back to the Early Eocene, ∼50 Mya.

While forcings and response are highly uncertain that far back in time, model/data comparison studies of the Early Paleogene (65 to 35 My before present) suggest a state-dependent ECS that increases with warming, both due to an increase in fast (i.e., Charney) climate feedbacks associated with cloud property adjustments and due to a nonlogarithmic increase in CO_2_ opacity (63). Shaffer et al. (64) used estimates of CO_2_ and global temperature change to estimate ECS both before and during the Paleocene/Eocene Thermal Maximum natural carbon release event ∼56 Mya. They estimate that ECS increased from a range of 3.3 to 5.6 °C to a range of 3.7 to 6.5 °C. Comparing these estimates with ECS estimates from the LGM and modern era suggests a significant increase in ECS with warming for greenhouse climates relative to colder climates.

Sherwood et al. (65) employed a Bayesian statistical approach to combine various lines of paleoclimate evidence in an attempt to reduce the current uncertainty range in ECS. They produced a revised likely (66% probability) “robust” range of 2.3 to 4.5 °C, reduced relative to the canonical 1.5 to 5 °C range cited earlier, and a further reduced “very likely” (95% probability) range of 2.0 to 5.7 °C. The strongest constraints at the upper end of the range, in their analysis, come from paleoclimate evidence from cold climates. That finding seems to be contradicted by evidence cited above for substantially higher sensitivities from hothouse climates of the distant past. Neither the cooling during the largest volcanic eruptions of the CE nor the cooling during the LGM can provide any constraint on feedback processes that are specific to hothouse climates. Even the most sophisticated statistical analysis cannot account for physical responses that lie outside the range of the data analyzed.

## Dangerous Warming

One uncertainty in evaluating the carbon budget left for avoiding critical warming thresholds such as the 2 °C and (aspirational) 1.5 °C warming limits adopted by the Paris climate accord involves the definition of the preindustrial baseline with respect to which warming is measured. Many studies have, for simplicity, adopted a late nineteenth century (e.g., 1850 to 1900) baseline since a reliable global surface temperature record is only available back to the mid-nineteenth century (66). However, fossil fuel burning and the rise in global CO_2_ concentrations began in the eighteenth century, and models predict that some anthropogenic greenhouse warming had occurred prior to the mid-nineteenth century (67). Given that the warming recorded by the instrumental record is already ∼1.2 °C, even a 10th of a degree Celsius has a large impact on how close we are to the 1.5 °C (or 2 °C) threshold and the carbon budgets left for avoiding those thresholds.

Given the uncertainties that exist in proxy reconstructions of global mean temperature during the CE (refer back to Fig. 1), these reconstructions provide relatively little constraint on preinstrumental warming. Climate model simulations, on the other hand, can provide more precise estimates of that warming. Schurer et al. (67) use the CMIP5 Last Millennium simulations to estimate how much anthropogenic warming had occurred prior to the late nineteenth–century period typically used to define the preindustrial baseline, finding evidence for anywhere from 0.1 to 0.2 °C additional warming, depending on the precise preindustrial time period used since there are centennial-scale preindustrial temperature fluctuations driven by natural (primarily solar and volcanic) radiative forcing. Taking into account this additional preinstrumental warming, Schurer et al. (67) estimate as much as a 40% reduction in the carbon budget available for avoiding 2 °C (and even greater reduction in the carbon budget for 1.5 °C). Along with other considerations, including how surface air temperature and SSTs are blended in calculating global mean temperature in models and observations and how instrumental and model temperature series are merged (68), such technical considerations demand greater precision in how warming targets and carbon budgets are defined by policy makers and other stakeholders.

## Looking Forward

The study of the CE can inform many of the key scientific questions that remain regarding climate dynamics and climate change. The large-scale warming trend of the past century is seen to be unprecedented in millennia (and likely even a longer time frame), confirming the unprecedented nature of human-caused climate change. Past relationships between natural solar and volcanic forcing hint at potential dynamical responses to human-caused warming (e.g., regional responses related to modes of variability, such as El Niño, the Asian summer monsoon, and the Atlantic conveyor belt ocean circulation, that remain uncertain). As these responses are likely to influence many key regional climate change impacts, it is critical to better understand them.

The preindustrial CE can also afford us an expanded view of natural climate variability. Analyses of the past millennium, for example, are seen to cast doubt on the existence of AMO-like internal multidecadal oscillations that have been invoked to argue against an impact of anthropogenic climate change on key climate impacts such as the observed increase in Atlantic hurricane activity. These analyses furthermore suggest limited potential for long-range climate predictability through initialized model prediction beyond the seasonal predictability afforded by ENSO.

Finally, studies of the CE inform important climate policy assessments. These include the evaluation of the increased coastal risk from sea-level rise and tropical storm intensification, the estimation of climate sensitivity to greenhouse gas increases, and estimates of the carbon budget remaining for keeping warming below critical 1.5 and 2.0 °C planetary danger limits.

What is the path forward to more confident insights? It is a truism that better models, more and higher-quality paleoclimate proxy data, and more careful comparisons of the two can yield more confident inferences. However, it is helpful to be more precise than that. Clearly, as we have seen, volcanic forcing plays a critical role in forced climate change during the CE, and yet, the estimates of the forcing remain widely variable (69). Efforts to reduce that uncertainty would clearly pay dividends, but so would efforts to expand and diversify the available networks of high-resolution proxy given the potential biases and limitations in dating, interpretation, and climate signal sensitivity that are specific to individual proxy types such as tree rings, corals, and ice cores.

Equally important, however, are current limitations in climate models used in Last Millennium experiments (e.g., CMIP5 and now CMIP6). As we have seen, ENSO-related dynamics play a particularly important role in regional climate responses. Yet, there is reason to believe that most current generation climate models may not exhibit the correct response of those dynamics to forcing. Furthermore, as noted earlier, potentially important AO/NAO responses to solar forcing require interactive ozone photochemistry, which has not been incorporated into most model simulations including the CMIP5 Last Millennium simulations (16).

Of course, limitations in current generation climate models lead not only to uncertainties in forced responses but internally generated variability as well. The possibility cannot be ruled out, for example, that the absence of AMO-like climate oscillations in current generation coupled models (35, 37), rather than indicating the absence of such oscillations in the real world, reflects a limitation in the representation of oceanic boundary currents, gyre and overturning circulations, and/or surface ocean–atmosphere coupling.

One particularly promising path forward combines the multiple sources of information we have—paleoclimate proxy data, models, and forcing estimates—in the form of data assimilation experiments. These experiments can be used to provide better constraint on key parameters of the climate system (70). More often, they are used to merge proxy data and model simulations in the process of reconstructing past climate fields (71–75).

Earlier proxy data assimilation efforts sought to reduce computational demand by employing “particle filter” approaches to assimilation with climate models of intermediate complexity (71, 72). More recent efforts such as the Last Millennium reanalysis project (73–75) have made use of long-term forced coupled model integrations (CMIP5 Last Millennium experiments). However, as always, caveats and limitations must be taken into account. The final product is no better than the models, data, and forcings that go into it, and the previously discussed limitations in each must be kept in mind.

In data assimilation, the climate physics of the model is in essence used to fill in missing information, a form of “smart interpolation” that yields complete climate fields. However, the accuracy of these reconstructed fields is limited by the ability of the models to reproduce real-world ocean and atmospheric circulation patterns. When the patterns present in the proxy data do not fit with the patterns produced by the models, the assimilation product represents an imperfect compromise between the conflicting sources of information (70), smearing out and distorting any real-world climate features. A specific source of concern is the fact, discussed earlier, that current climate models do not appear to reproduce SST gradients in the tropical Pacific during the historical era and do not match longer-term trends in those gradients inferred from paleoclimate data. Data assimilation is unlikely to resolve such fundamental discrepancies and may simply obscure what is actually happening.

There are of course the limitations in the underlying proxy data themselves and how they are assimilated into the models. Past studies have generally (70–74) assumed simple linear relationships between proxy data and the model variables (temperature, precipitation, upwelling, etc.) they are purported to represent. However, as discussed earlier, there are open questions about tree rings and their ability to record the largest volcanic cooling events; complications in tree rings, corals, and ice cores due to mixed precipitation and temperature signals; and the problem of threshold response limits of climate proxies. Such complications could in principle be accounted for through the use of nonlinear regression methods such as neural nets or the use of forward models connecting the proxy data to the target model variables that account for nonlinearities and threshold response limits. One particularly attractive prospect is the availability of interactive tools that allow users to diagnose the impact of adding a particular proxy record or set of proxy records to the data assimilation product, building on earlier efforts to investigate proxy sampling strategies (76, 77).

As important as paleoclimate is to addressing fundamental questions today regarding climate science, climate impacts, and climate policy, we must make sure not to overpromise what it can provide, particularly when there is the potential for findings to be used in crafting climate policy. Consider, for example, the previous discussion of ECS. While paleoclimate data might provide solid constraints on the “low end” of the ECS spectrum, there is reason to be skeptical about efforts to narrow the high end of the spectrum based primarily on cold climate constraints, especially when work focused specifically on past hothouse climates suggests substantially higher climate sensitivities. What might be more relevant to projected future climate change is the warm climate, transient response (TCR) to increasing greenhouse gas concentrations.

We should furthermore not brush real discrepancies between models and proxy observations “under the rug.” We should not (78), for example, dismiss systematic underestimates of reconstructed responses to forcing as simply an artifact of presumed errors in the models or the forcing estimates. There is good reason to suspect potential systematic biases and limitations in the underlying proxy data themselves.

It is important to recognize the potential limitations of paleoclimate studies in addressing some outstanding questions. There is no shame in the paleoclimate research community acknowledging that paleoclimate studies cannot address all outstanding questions regarding climate dynamics, climate variability, and climate change. Paleoclimate evidence should instead be viewed as providing one very valuable source of information that, combined with other sources, can yield a fuller understanding and appreciation of the climate system. Constructive feedback from other sectors of the climate research community should be taken in good faith and recognized as critical to continued progress in the field. Two decades after my coauthors and I published the hockey stick curve, I look forward to both observing and participating in that further progress.

## Data Availability

There are no data underlying this work.
